# PB1-F2 Attenuates Virulence of Highly Pathogenic Avian H5N1 Influenza Virus in Chickens

**DOI:** 10.1371/journal.pone.0100679

**Published:** 2014-06-24

**Authors:** Olivier Leymarie, Carissa Embury-Hyatt, Christophe Chevalier, Luc Jouneau, Marco Moroldo, Bruno Da Costa, Yohannes Berhane, Bernard Delmas, Hana M. Weingartl, Ronan Le Goffic

**Affiliations:** 1 Unité de Virologie et Immunologie Moléculaires, Unité de Recherche UR892, Institut National de la Recherche Agronomique, Jouy-en-Josas, France; 2 National Centre for Foreign Animal Disease, Canadian Food Inspection Agency, Winnipeg, Manitoba, Canada; 3 Centre de Ressources Biologiques pour la Génomique des Animaux Domestiques et d'Intérêt Economique, Institut National de la Recherche Agronomique, Jouy-en-Josas, France; 4 Department of Medical Microbiology, University of Manitoba, Winnipeg, Manitoba, Canada; Centre of Influenza Research, The University of Hong Kong, Hong Kong

## Abstract

Highly pathogenic avian influenza virus (HPAIV) is a permanent threat due to its capacity to cross species barriers and generate severe infections and high mortality in humans. Recent findings have highlighted the potential role of PB1-F2, a small accessory influenza protein, in the pathogenesis process mediated by HPAIV in mammals. In this study, using a recombinant H5N1 HPAIV (wt) and its PB1-F2-deleted mutant (ΔF2), we studied the effects of PB1-F2 in a chicken model. Unexpectedly, when using low inoculation dose we observed that the wt-infected chickens had a higher survival rate than the ΔF2-infected chickens, a feature that contrasts with what is usually observed in mammals. High inoculation dose had similar mortality rate for both viruses, and comparison of the bio-distribution of the two viruses indicated that the expression of PB1-F2 allows a better spreading of the virus within chicken embryos. Transcriptomic profiles of lungs and blood cells were characterized at two days post-infection in chickens inoculated with the wild type (wt) or the ΔF2 mutant viruses. In lungs, the expression of PB1-F2 during the infection induced pathways related to calcium signaling and repressed a large panel of immunological functions. In blood cells, PB1-F2 was associated with a gene signature specific for mitochondrial dysfunction and down-modulated leucocytes activation. Finally we compared the effect of PB1-F2 in lungs of chickens and mice. We identified that gene signature associated to tissue damages is a PB1-F2 feature shared by the two species; by contrast, the early inhibition of immune response mediated by PB1-F2 observed in chickens is not seen in mice. In summary, our data suggest that PB1-F2 expression deeply affect the immune response in chickens in a way that may attenuate pathogenicity at low infection dose, a feature differing from what was previously observed in mammal species.

## Introduction

Since 1997, H5N1 highly pathogenic avian influenza virus (HPAIV) is an omnipresent public health threat [1]. This concern is based on capacity of such virus to cross the species barrier from their avian hosts to humans. Waterfowl and shorebirds constitute the natural hosts of H5N1 HPAIV; infected wild aquatic birds usually develop relatively mild symptoms and limited disease. In ducks, infection is usually asymptomatic [2], [3]. Nevertheless, when the virus is transmitted to poultry, mortality rate can reach 100%. The ability of H5N1 HPAIV to cross species barriers and spread to humans has been reported in 15 countries and confirmed by World Health Organization (WHO). The total number of cases confirmed by WHO is 650, with case fatality rate of 60% [4]. However, seroprevalence study among poultry workers suggested that H5N1 HPAIV could also cause infections with only mild symptoms in humans [5]. Human-to-human H5N1 HPAIV transmission has not been described yet, but recent works designed to address the questions of transmissibility and adaptation of H5N1 HPAIV to the human host identified important determinants in the HA-encoding segment [6], [7].

Influenza A virus belongs to the *Orthomyxoviridae* family, and its genome consists of eight negative strand RNA segments encoding up to 14 proteins [8], [9]. Viral determinants conferring host adaptation can be acquired through segment exchange between strains. This gene reassortment ability provides genome plasticity facilitating the crossing of species barriers [10], [11]. The PB1-encoding segment from the H1N1 influenza viruses responsible for the 1957 and 1968 pandemics was shown to have an avian origin [10]. This segment was also demonstrated to exert an important role in the pathogenesis mediated by the 1918 pandemic influenza virus [12]. In addition to the PB1 component of the polymerase complex, this segment 2 also encodes the N40 protein, a N-truncated version of PB1 lacking transcriptase function, and PB1-F2, a pro-apoptotic protein [13], [14]. PB1-F2 drew attention in recent years since a number of reports associated its expression with an increase of virus pathogenicity [15]–[21]. When looking at the prevalence of a functional PB1-F2 in strains of various origins, its expression appears unequally distributed: for example 96% of avian strains encodes a full-length PB1-F2 while only 7% of human H1N1 isolates express a functional PB1-F2 [22], [23]. Thus, several reports suggest that the loss of a functional PB1-F2 could be beneficial for the virus when it crosses the species barrier to spread through humans [22], [24]–[26]. Consequently, the loss of PB1-F2 could be an adaptation process of the virus to mammalian hosts in order to confer optimized replicative efficiencies and viral fitness [27]. On the contrary, PB1-F2 from a highly virulent avian strain could be acquired by a circulating human strain through segment exchange, and then contribute to increased virulence associated with seasonal influenza viruses.

PB1-F2 was first described in 2001 [13]. This 90 amino-acid long protein is encoded by an alternative open reading frame (ORF) overlapping the PB1 ORF. In mammals, PB1-F2 can induce apoptosis of immune cells [13] and promote inflammation in a strain-dependent manner [17], [19], [27], [28]. PB1-F2 triggers apoptosis by disturbing the mitochondrial membrane potential, an event that can lead to cytochrome c release and subsequent activation of caspases [29]. Inflammation induction is triggered by an exacerbation of the NF-κB transcription factor activity [19], [30]. The molecular basis of this exacerbation is currently unknown but the propensity of PB1-F2 to form β-sheet structures and amyloid fibers are suspected to play an important role in this process [31]. PB1-F2 has also been described to up-regulate the viral polymerase activity [32], but this property is strain-specific and has no impact on the pathogenesis [33].

In mammals, PB1-F2 behaves as a virulence factor [15]. A recombinant WSN/1933 (H1N1) mutant lacking PB1-F2 has been shown to be less virulent than its wild-type (wt) counterpart [19]. Such effects on the mortality are not always visible, especially when highly pathogenic H5N1 strains are studied due to the extreme 50% lethal doses (LD50) of these viruses [34]. However, in mice models, the high LD50 can be by-passed using Mx+/+ transgenic mice [20]. Varga and co-workers described the inhibition of type I interferon (IFN) by PB1-F2; they showed that PB1-F2 antagonizes the function of MAVS through disruption of the mitochondrial membrane potential [18], [35], [36]. On the other hand we observed an exacerbation of the IFN expression when PB1-F2 was expressed during WSN/1933 infection [30]. This feature correlates well with the pro-inflammatory properties of PB1-F2. The sequence polymorphism of PB1-F2 that dictates the pro-inflammatory properties of PB1-F2 may explain the observed differences [28].

In this study, we compared the pathological process mediated by a H5N1 HPAIV unable to express PB1-F2 with its PB1-F2-expressing counterpart in chickens to elucidate the role of PB1-F2 in an avian host. Survival curves, bio-distribution and host responses to both viruses were analyzed in order to determine and analyze the impact of PB1-F2 expression in this host. Unexpectedly, and in contrast to what is observed in mammals, PB1-F2 attenuates virulence in chicken. Using functional genomics tools, we delineated the impact of PB1-F2 in the lungs of infected chickens. Finally we compared these effects with what we previously observed in mouse infected with the same H5N1 HPAIV couple [34].

## Results

### Viral distribution of wild-type and PB1-F2 deleted viruses in chicken embryos

**Figure 1:**
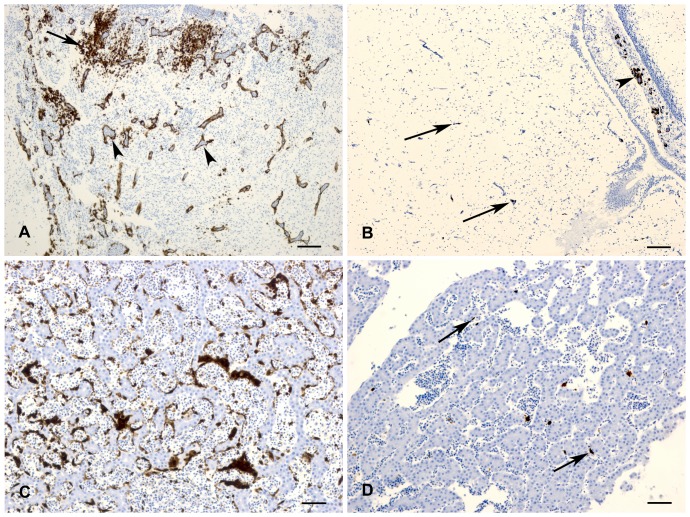
Immunohistochemical detection of influenza A antigen in chicken embryos at 24-inoculation. (A) Brain; Wt group. Extensive immunostaining was observed in endothelial cells (arrowheads) and well as multifocal areas of brain parenchyma involving neurons (arrow). (B) Brain; ΔF2 group. There were occasional small foci of endothelial (arrows) and parenchymal (arrowhead) staining. (C) Liver; Wt group. There is extensive immunostaining of sinusoidal lining cells as well as scattered hepatocytes. (D) Liver, ΔF2 group. There was mild immunostaining of individual sinusoidal lining cells (arrows).

The recombinant influenza A/duck/Niger/2090/2006(H5N1) (herein named Nig06) and its PB1-F2 knocked out counterpart (named ΔF2) were previously produced [34]. To study the effects of PB1-F2 expression in the chicken host, we first performed histological investigations to characterize the bio-distribution of Nig06 and ΔF2 viruses within chicken embryos. Examination of embryos at 24 hours post inoculation by immunohistochemistry showed that the viral antigen was more widespread in the wt group as compared to the ΔF2-virus group. In examined tissues of most of the embryos including the brain, heart, spleen, liver, lung, kidney, spinal cord, bone and skeletal muscle, there was weak to moderate immunostaining in the ΔF2 group whereas extensive to widespread immunostaining was observed in the wt group (Fig. 1). In both groups antigen was primarily observed in endothelial cells in all tissues. Some spread to surrounding parenchyma including hepatocytes, neurons, skeletal muscle, cardiomyocytes and pneumocytes was observed in both groups but was more pronounced in the wt group. In both groups there was consistently strong immunostaining in the chorioallantoic membrane. At the 18 and 48 hour timepoints post-inoculation, there were no differences observed between the two groups. In summary, these data suggest that PB1-F2 expression may accelerate the systemic spreading of the virus, as previously described [20].

### Mortality associated with PB1-F2 from Nig06

**Figure 2:**
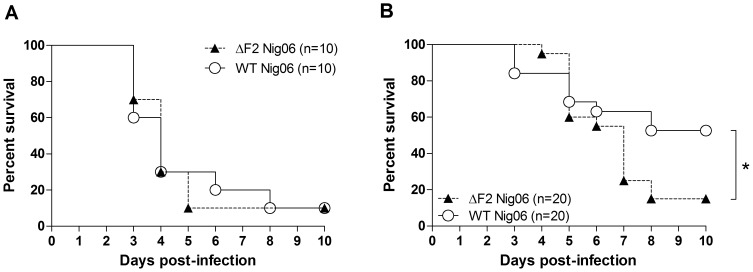
Characterization of the mortality induced by the wt and ΔF2 Nig06 HPAIV. Survival curves of chickens infected with wt or ΔF2 Nig06 virus. The chickens were inoculated via nares with 0.5 PBS containing 1000 (A) or 100 (B) PFU. The chickens were observed for 10 days after challenge.

To determine the role of PB1-F2 in highly pathogenic AIV pathogenesis, we first aimed to characterize the impact of PB1-F2 on the mortality in adult 8 weeks old white leghorn chickens. Chickens were challenged using 1000 plaque forming units (PFU) of wt Nig06 (n = 10) or ΔF2 Nig06 (n = 10). As shown in Fig. 2A, this amount of virus killed 90% of the chickens. Death of the animals occurred between days 3 and 8 post-infection (pi) and the mean time of death is 4 days pi. No differences could be observed between the 2 types of viruses. Remarkably, we found that wt Nig06-infected chickens (n = 20) had a higher survival rate than ΔF2 Nig06-infected chickens (n = 20): 50% *vs.* 15% respectively in survival experiments using a lower viral dose of 100 PFU (Fig. 2B). The chicken LD_50_ was estimated to be of 10^2.05^ PFU for the wt Nig06, and of 10^1.62^ PFU for the ΔF2 Nig06. These results suggest that the expression of PB1-F2 in birds infected with low dose of HPAIV could be beneficial for the avian host survival.

### Replication and shedding of wt and ΔF2 Nig06

**Figure 3:**
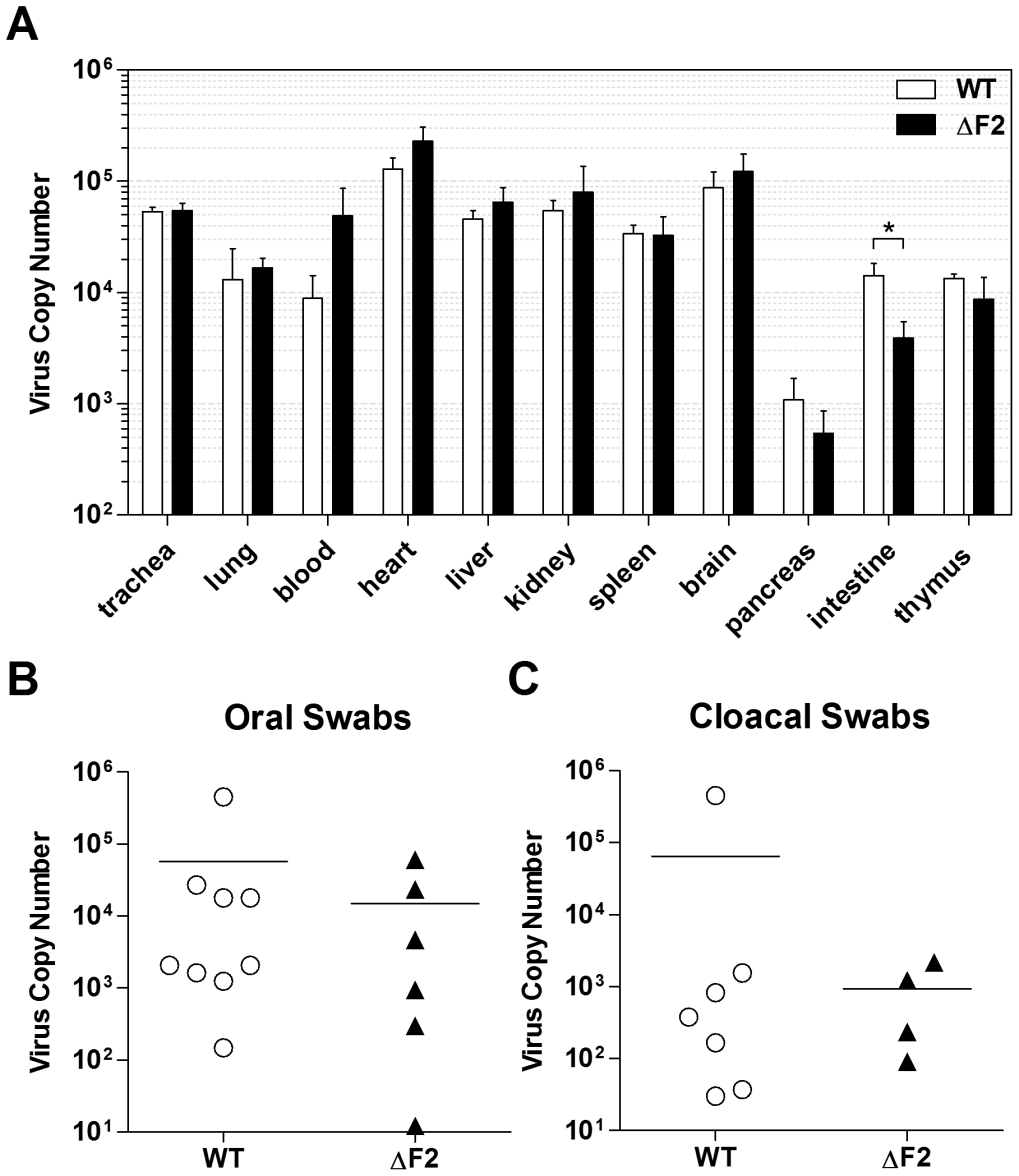
Tissue distribution and shedding of the wt and ΔF2 Nig06 HPAIV. (A) Viral titers of the indicated organs were measured by qRT-PCR at day 2 pi with 1000 PFU of wt or ΔF2 Nig06 virus. (B) Oral and (C) cloacal swabs were collected at day 2 pi to detect and quantify viral shedding using qRT-PCR.

To further characterize the role of PB1-F2 in the pathogenic process exerted by the viral infection, we investigated whether PB1-F2 could influence the spreading of the virus by quantifying the tissue distribution of the virus using qRT-PCR after 2 days pi (Fig. 3A). The amounts of viral copy number of wt and ΔF2 viruses were roughly the same in trachea, lung, spleen and thymus tissues. However, in the blood of ΔF2-infected chicken, although the difference is not statistically significant (p = 0.17), we observed a higher amount of virus as compared to wt-infected chicken virus. Such differences are also observed in heart, liver, kidney and brain but to a lesser extent. In contrast, in the pancreas (not statistically significant) and in the intestine, we detected a higher amount of viral copies in the wt-infected chicken. Importantly, the difference observed in the intestine is statistically significant (p<0.05), and suggests that PB1-F2 could influence the enteric tropism of the virus, though, as the difference between the two conditions is less than one log viral copy number, this result could be statistically significant but biologically non relevant. We next compared the shedding capacities of the wt and the ΔF2 viruses. Oropharyngeal and cloacal swabs were taken at day 2 pi for virus copy number quantification. The analysis of swabs from wt-infected chickens revealed a higher number of positive samples (7/10 vs. 4/10 for the ΔF2 group), yet, when looking at the amount of viral RNA detected within the positive individual swabs, no differences could be evidenced between the two types of infection (Fig. 3B and 3C). In order to confirm the qRT-PCR virus RNA quantification data, we attempted virus isolation on oral swabs by plaque assay method to quantify the virus shedding (Figure S1).

### Impact of PB1-F2 on the expression profiles of representative genes of the host response

**Figure 4:**
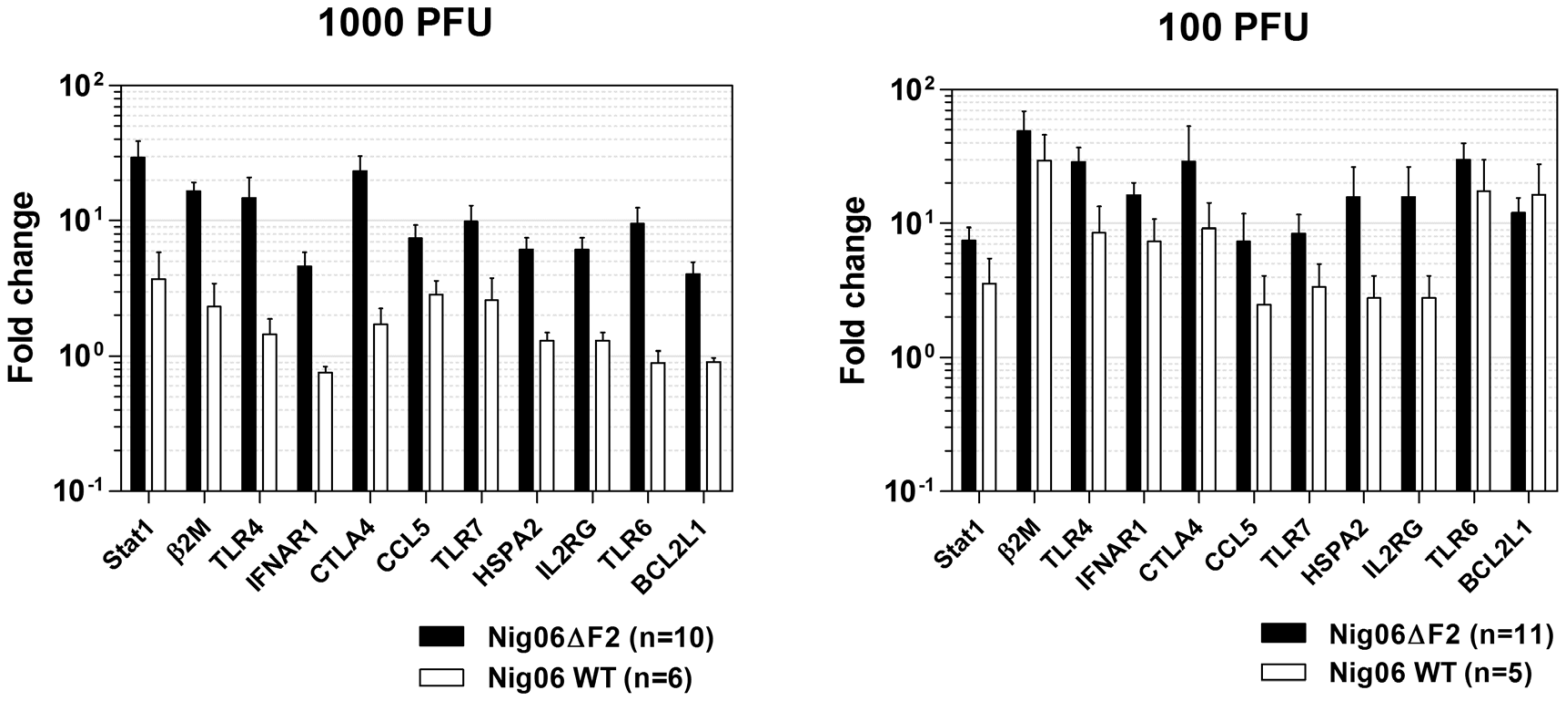
Impact of PB1-F2 on expression profiles of several genes representative of the host response. Total RNA isolated from lungs of chickens infected with 1000PFU (A) and (B) (day 2 pi) were reverse-transcribed and used to quantify expression of several host response markers by qPCR: signal transducer and activator of transcription 1 (STAT1, Gene ID: 424044), beta-2-microglobulin (β2M, Gene ID: 414830), toll-like receptor 4 (TLR4, Gene ID: 417241), interferon (alpha, beta and omega) receptor 1 (IFNAR1, Gene ID: 395665), cytotoxic T-lymphocyte-associated protein 4 (CTLA-4, Gene ID: 424106), chemokine (C-C motif) ligand 5 (CCL5, Gene ID: 417465), toll-like receptor 7 (TLR7, Gene ID: 418638), heat shock 70 kDa protein 2 (HSPA2, Gene ID: 423504), interleukin 2 receptor gamma (IL2RG, Gene ID: 395199), toll-like receptor 6 (TLR6, Gene ID: 771173), and B-cell CLL/lymphoma 2-like 1 (BCL2L1, Gene ID: 373954). Gene expressions were normalized with the β-actin gene (Gene ID: 396526) expression level and presented as fold increase relative to mock-treated chickens. Data are means ± SEM obtained from the indicated number of chickens.

Prior to characterization of the global transcriptome of the infected-chickens, we first explored the impact of PB1-F2 on the host response by using qRT-PCR assays. We focused on several gene representatives of the inflammatory and immune responses: STAT1, β2M, TLR4, IFNAR1, CTLA-4, CCL5, TLR7, HSPA2, IL2RG, TLR6 and BSL2L1. The two inoculum doses were then compared (Fig. 4). Surprisingly, despite the outcome of the survival curves, the differences between wt- and ΔF2-infected chickens were larger and statistically more significant in the 1000 PFU group (p value: 0.0006 *vs.* 0.0331). Consequently, the 1000 PFU inoculum dose was chosen for further global transcriptomic analysis.

### Overview of differences in gene expression following infection with wt or ΔF2 Nig06

**Figure 5:**
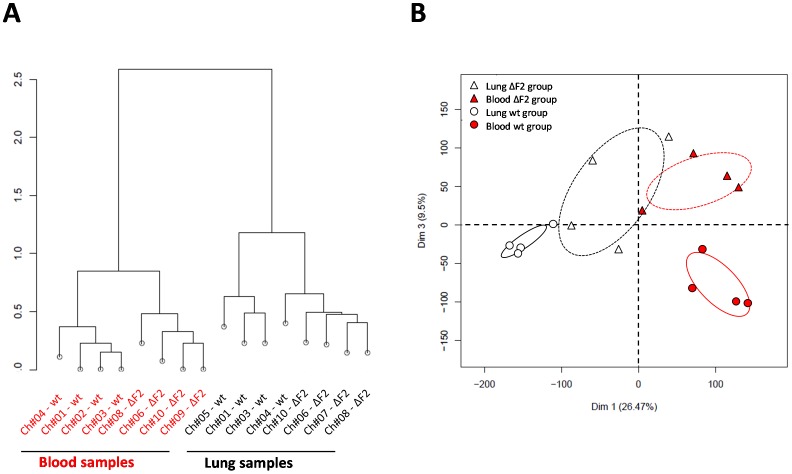
Correlation clustering and principal component analysis of microarray data. The expression value for each gene from lung and blood tissues of wt and ΔF2-infected chickens were used for the analysis. (A) The expression distance was measured between all pairs of arrays and represented as a tree. (B) The principal component analysis of the expression values was made to represent the variability between arrays. X-axis represents the first dimension (Dim 1, 26.47% of the variance) which separates the tissue samples (lung *vs.* blood), y-axis represent the third dimension (Dim 3, 9.5% of the variance) which separates the viral infections (wt *vs.* ΔF2). Clusters of samples on the PCA plot present a strong correlation of expression signals.

To determine how PB1-F2 modulates virus pathogenicity, we investigated the overall host response to wt- and ΔF2-Nig06 infection. Microarray analyses of RNA samples extracted from lungs and blood samples of wt- or ΔF2-infected chicken were carried out. Two groups of 5 White Leghorns chickens were intranasally infected using 1000 PFU of each virus. After 2 days pi, blood samples were collected; chickens were euthanized at the same day and lungs were collected. Total RNA were extracted, processed and analyzed using the Agilent Chicken (V2) Gene Expression Microarray (4×44 K). Each probe signals were normalized and statistically treated as described in the materials and methods section. To identify outliers arrays, hierarchical correlation clustering (uncentered with average linkage) of the entire set of samples was carried out. As a consequence, one chicken was excluded in each group of the functional analysis. The 8 remaining chicken specimens showed a strong “compartment” effect: lungs and blood samples displayed a clear distinct clustering (Fig. 5A). Remarkably, within each “compartment” cluster, the 2 viral infections also constitute 2 separate clusters, indicating a clear effect of PB1-F2 during the Nig06 infection of chickens. In order to illustrate the global variance by which different sample sets correlate, arrays data from each samples were subjected to principal component analysis (Fig. 5B). As for the hierarchical correlation clustering, the 4 sets of samples clustered in distinct localized areas, indicating different gene expression profiles in lung and blood in response to wt or ΔF2 viruses. Collectively, these data indicate a potent effect of PB1-F2 during the chicken response to Nig06 infection, and also a tissue specific-effect of PB1-F2.

### Analysis of the impact of PB1-F2 expression in Nig06-infected chickens lungs

**Figure 6:**
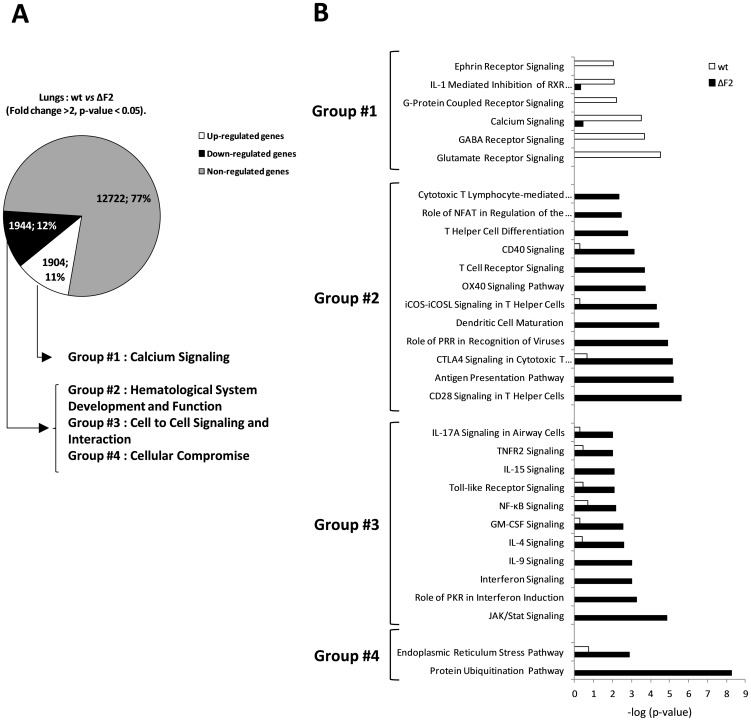
Global analysis of PB1-F2-dependant gene expression changes in the lungs of influenza infected chickens at day 2 pi. (A) Distribution of genes differentially regulated between wt- and ΔF2-infected chicken lungs. Major functions according to GO annotated function are indicated for each category of regulated genes. (B) Canonical pathways associated to differentially expressed genes were compared using Ingenuity Pathways Analysis. Canonical pathways are ranked using the p-value obtained using the right-tailed Fisher's exact test, it represents the probability of each biological function to be involved in the group of analyzed genes.

**Figure 7:**
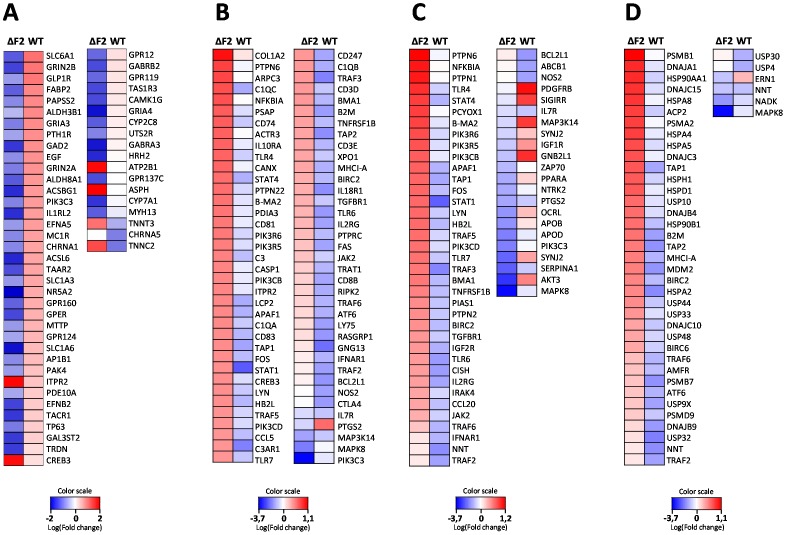
Heat maps of selected genes in the lungs of ΔF2- and wt-infected chickens at day 2 pi. Genes shown in red are upregulated and genes in blue are downregulated. Data are expressed in Log base 2 ratio. (A) Heat map of genes related to “Calcium Signaling pathway”. (B) Heat map of genes related to “Hematological System Development and Function”. (C) Heat map of genes related to “Cell to Cell Signaling and Interaction”. (D) Heat map of genes related to “Cellular Compromise”.

The functional consequences of PB1-F2 expression during Nig06 infection of lungs was addressed by analyzing the functions associated with the differentially expressed genes. Regulated genes in the group of wt-infected chickens were directly compared to regulated genes from the group of ΔF2-infected chicken to generate 2 sets of genes: genes up-regulated and genes down-regulated in presence of PB1-B2. We selected genes that were at least 2 fold different in their expression between wt- and ΔF2-infected chicken (adjusted p-value<0.05). Figure 6A represents the distribution of the genes, among the 3848 regulated genes, half of them appear up-regulated in the presence of PB1-F2 and half of them appear down-regulated. We then explored the functional consequences of PB1-F2 expression by using ontological annotations of the differentially expressed genes. The set of genes up-regulated during wt Nig06 infection revealed a strong association of PB1-F2 to “Calcium Signaling” pathways: Glutamate Receptor Signaling, GABA Receptor Signaling, G-Protein Coupled Receptor Signaling (Fig. 6B). This suggests that PB1-F2 could disturb intracellular calcium stores and interfere with pathways in which calcium is involved. Logically, when looking at the heat map representative of this pathway (Fig. 7A), we observed that most of the genes were down-regulated in the absence of PB1-F2 except few genes playing important role in calcium homeostasis: ITPR2, ATP2B1, ASPH and CREB3. ITPR2 and ATP2B1 are two Ca^2+^ pumps localized within the endoplasmic reticulum (ER) [37], [38]. ASPH is an aspartate-hydroxylase regulating the Ca^2+^ release from the ER [39]. CREB3 is a ER-bound transcription factor activated by Ca^2+^ signaling and involved in inflammatory gene expression [40]. Overall, the analysis of this group of genes revealed that PB1-F2 expression modulates Ca^2+^ signaling pathways within the lungs of infected chicken.

The set of genes down-regulated by PB1-F2 (*i.e.* up-regulated in the ΔF2-infected chicken lungs) can be divided into 3 different main functions: “Hematological System Development and Function”, “Cell to Cell Signaling and Interaction” and “Cellular Compromise” (Fig. 6A). The cluster of genes associated with “Hematological System Development and Function” (group #2, Fig. 6A and 6B) illustrates the strong impact of PB1-F2 on immune cells. PB1-F2 expression suppressed the activation of multiple pathways essential for the mobilization and activation of dendritic cells and lymphocytes: CD40, OX40, iCOS, CTL4 and CD28 signaling pathways. This strong effect is linked to the regulation of the group #3 of genes which is involved in “Cell to Cell Signaling and Interaction”. It is composed of genes regulating the signal transduction of several cytokines including NF-kB and interferon signaling pathways. Heat maps of representative genes of these 2 main functions are shown in figures 7B and 7C.

The expression of PB1-F2 also down-regulate a cluster of genes functionally associated to “cellular compromise”. Two canonical pathways are linked to these genes: “ER Stress Pathway” and ”Protein Ubiquitination Pathway”. These two functional categories illustrate the virulence factor properties of PB1-F2 during influenza virus infection. As shown in figure 7D, we found that the expression of PB1-F2 during the infection represses the entire gene cluster representative of the two pathways with the exception of ERN1. Interestingly, ERN1 (also known as IRE1) is a transmembrane protein resident of the ER which is implicated in the sensing of unfolded proteins in the lumen of the ER. Activation of ERN1 leads to a potent transcriptional response triggering growth arrest and apoptosis [41]. Collectively, these transcriptomic data on the lung response associated to PB1-F2 revealed a potent effect of PB1-F2 on the host response within chicken airways, and particularly on genes involved in Ca^2+^ homeostasis and ER stress pathways.

### Analysis of the impact of PB1-F2 expression in blood of Nig06-infected chickens

**Figure 8:**
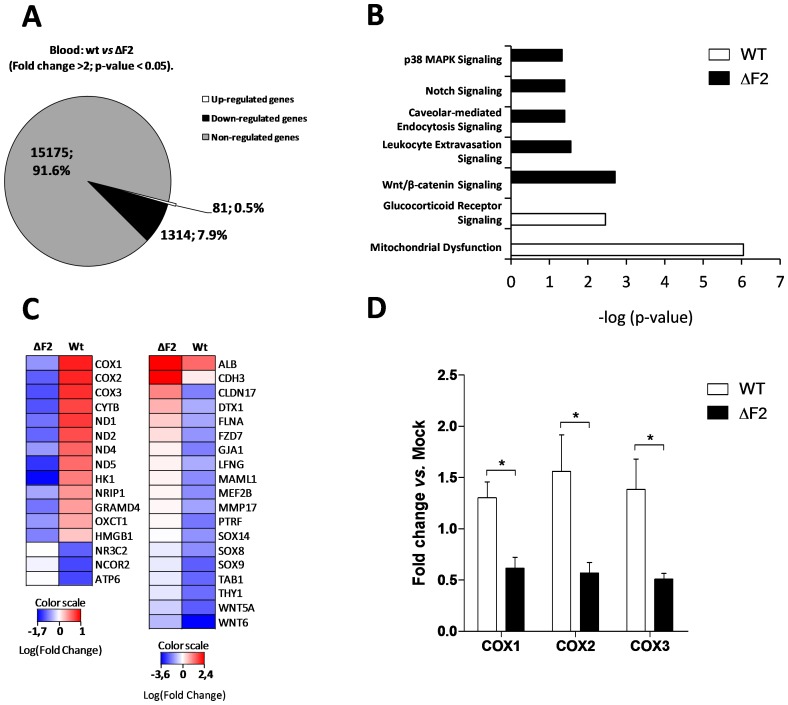
Global analysis of PB1-F2-dependant gene expression changes in the blood of influenza infected chickens. (A) Distribution of genes differentially regulated between wt- and ΔF2-infected chicken blood samples. (B) Canonical pathways associated to differentially expressed genes were compared using Ingenuity Pathways Analysis. Canonical pathways are ranked using the p-value obtained with the right-tailed Fisher's exact test, it represents the probability of each biological function to be involved in the group of analyzed genes. (C) Heat maps of genes related to “Mitochondrial Dysfunction” (left panel) and to “Leukocyte activation pathways” (right panel). Genes shown in red are upregulated and genes in blue are downregulated. Data are expressed in Log base 2 ratio. (D) RT-PCR quantifications of COX1, COX2 and COX3 mitochondrial gene expressions in blood of wt- and ΔF2-infected chickens at day 2 pi. Gene expressions were normalized with the β-actin gene expression level and presented as fold increase relative to mock-infected chickens. Data are means ± SD of 4 chickens. *: p<0.05.

As illustrated in Fig. 2A, HPAIV are able to spread systemically in chicken. To gain insight into the functions of PB1-F2 beyond the respiratory tract, we studied the host response of chicken blood cells. RNA samples were collected before infection and at day 2 pi. Each RNA sample from blood of infected chicken was directly compared to RNA sample from the same chicken before infection using a dual-color hybridization design. Four chickens in each group (wt- and ΔF2-infected) were used to identify the genes activated during the host response. The PB1-F2-dependant gene profile in blood cells was very different from the profile obtained in lungs: the set of up-regulated genes was restricted to 81 genes, representing only 0.5% of the analyzed genes (Fig. 8A). The number of down-regulated genes was comparable to that observed in the lungs (7.9%). The 81 PB1-F2-up-regulated genes are strongly associated to “Mitochondrial Dysfunction” and “Glucocorticoid Receptor Signaling” pathways (Fig. 8B). The mitochondrial dysfunction pathway appears very interesting since PB1-F2 is mainly localized to the mitochondria [13], [42], [43] and is able to permeabilize mitoplasts (Christophe Chevalier, unpublished data). Among those 81 genes, a number of genes encoded by the mitochondrial genome are present (Fig. 8C, left panel). The relative levels of expression of 3 mitochondrial genes: *COX1*, *COX2* and *COX3* were confirmed in blood samples using quantitative PCR (Fig. 8D). The ontological analyses of down-regulated genes showed that PB1-F2 down-regulates activation of leukocytes (Leukocyte Extravasation Signaling, p38 MAPK Signaling, Wnt/β-catenin Signaling; Fig. 8B). A heat map of representative genes implicated in these biological processes is represented in fig. 8C (right panel).

Taken together, these blood cells transcriptomic data suggest a strong induction of mitochondrial genes transcription mediated by PB1-F2 and a down-regulation of leukocyte activation which could be due to an alteration of the mitochondria integrity. The loss of the mitochondrial membrane potential could also explain the inhibition of leukocytes activation as previously described [36].

### Comparison of Nig06 PB1-F2 functions in chicken and mouse infected lungs

**Figure 9:**
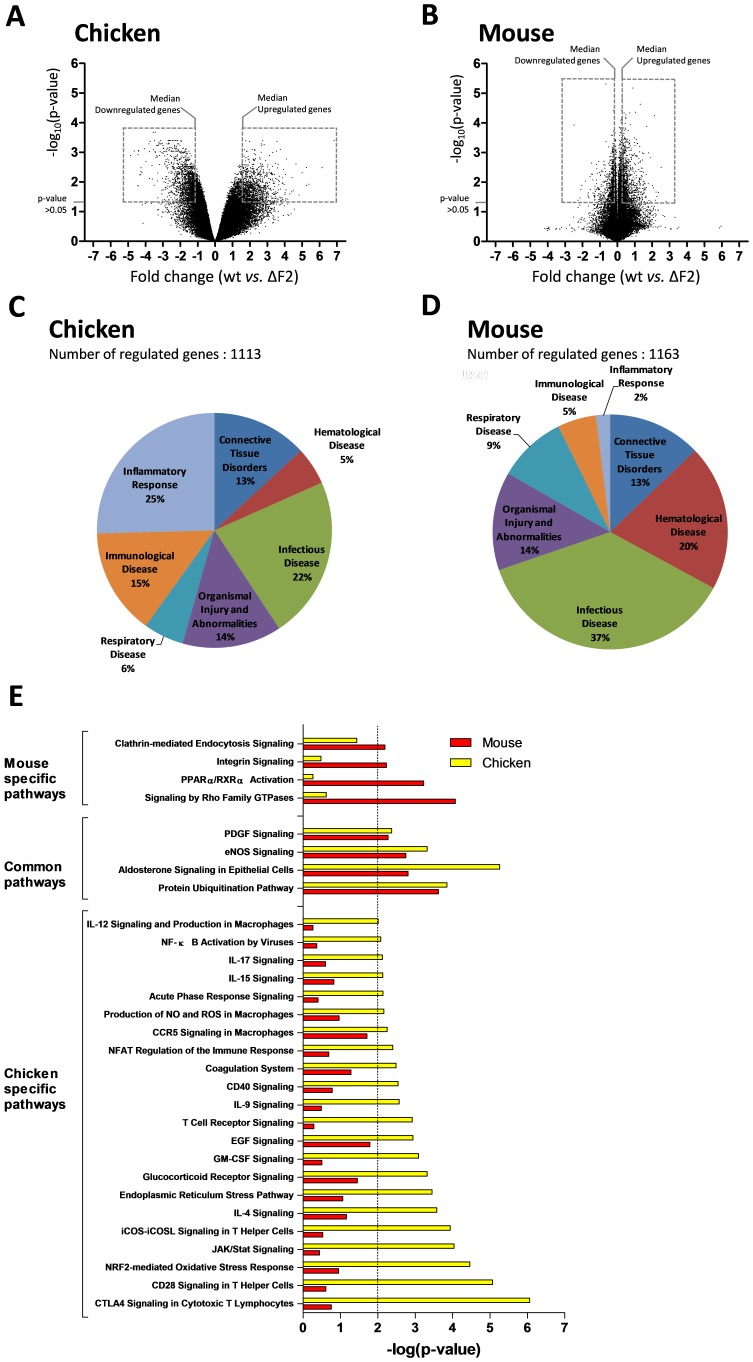
Comparison of Nig06 PB1-F2 impact on the lung host-response in chicken and mouse. (A) Scatter plot comparing wt-infected chickens vs. ΔF2-infected chicken lung transcriptomes at day 2 pi. Genes selected for ontological analysis are highlighted. (B) Scatterplot comparing wt-infected mice vs. ΔF2-infected mice lung transcriptomes at day 2 pi. (C) Distribution of major GO functions associated to genes regulated by PB1-F2 in infected chicken lungs. (D) Distribution of major GO functions associated to genes regulated by PB1-F2 in infected mouse lungs. (E) Canonical pathways associated to differentially expressed genes in chicken and mouse were compared using Ingenuity Pathways Analysis and ranked with the p-value obtained using the right-tailed Fisher's exact test.

**Figure 10:**
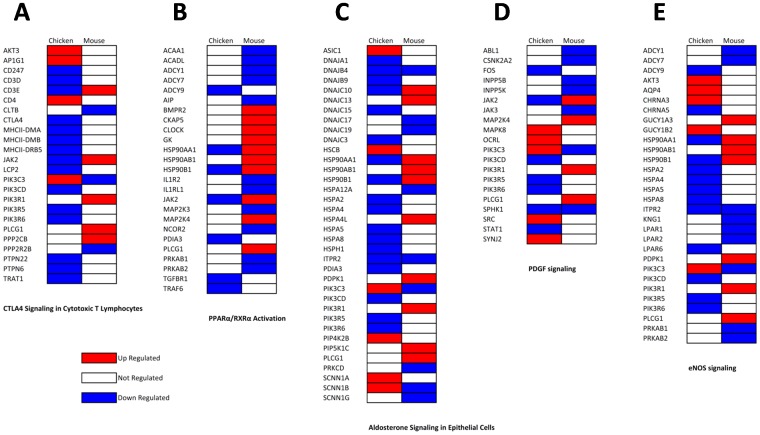
Heat maps of selected genes showing differential regulation in response to PB1-F2 in lungs of chicken and mouse at day 2 pi. Genes are representative of “CTLA4 Signaling in Cytotoxic T Lymphocytes” (A), “PPARα/RXRα Activation” (B), “Aldosterone Signaling” (C), “PDGF Signaling” (D) and “eNOS signaling” (E). Genes shown in red are up-regulated and those shown in blue are down-regulated in presence of PB1-F2.

Since the Nig06 is able to infect mammals and birds, we compared the host response associated with PB1-F2 in the lungs of mice and chickens after 2 days pi. We used the chicken data obtained in the present study and the mouse data published in a previous work [34]. We generated two sets of genes by directly comparing wt- and ΔF2-infected animals. The median up- and median down-regulated genes were selected using an adjusted p-value<0.05 (Fig. 9A and 9B), then we compared these two groups of genes in an ontological analysis. The functional classification of the genes regulated by PB1-F2 in chicken and mouse revealed major differences in the host response. In chickens, genes associated with “inflammatory response” and “immunological disease” correspond with 25% and 15% of the regulated-genes respectively, whereas in mice these functions are underrepresented with only 2% and 5% (Fig. 9C and 9D). When comparing the canonical pathways associated with the genes deregulated by PB1-F2 in both species, we identified host-specific PB1-F2 functions. As shown in Fig. 9E, in chicken, PB1-F2 regulated numerous inflammatory and immune pathways. Importantly, most of these pathways were down-regulated by the expression of PB1-F2 during the infection. For example, in chicken, the majority of the genes implicated in “CTLA4 Signaling in Cytotoxic T Lymphocytes” were down-regulated, suggesting that this specific function is down-modulated by PB1-F2 or that the cell type involved in this pathway is depleted by infection with PB1-F2 expressing virus (Fig 10A). On the contrary, in mouse, only few canonical pathways were regulated by PB1-F2 at this time-point. In particular, we found that PB1-F2 upregulated the expression of genes associated with “PPARα/RXRα Activation”, a pathway exerting anti-inflammatory functions (Fig. 10B). Presumably as a consequence of this, a delay in the triggering of the immune response is observed in mouse infected by the PB1-F2-expressing Nig06 virus [34]. Our data also indicate that PB1-F2 expression triggers host responses shared by avian and mammal hosts. Among these responses, we found “Aldosterone Signaling”, “PDGF Signaling” and “eNOS signaling” pathways which illustrate the damages caused by PB1-F2 within the epithelium, connective tissue and endothelium respectively (Fig. 10C-E).

In summary, PB1-F2 from Nig06 exerts different functions in mouse and chicken. In lungs of the avian host, PB1-F2 strongly decreases the inflammation response whereas its impact is mild in mice. However, the epithelial cells damage provoked by PB1-F2 expression appears to be a feature shared by both species.

## Discussion

Highly pathogenic H5N1 AIV infections among domestic poultry have become endemic in several countries in Asia and in Egypt [44]. An AIV is defined as “highly pathogenic” when it causes at least 75% mortality in 8-week-old naïve chickens intravenously infected [2], or if its hemagglutinin contains polybasic cleavage site [45]. Most highly pathogenic AIV provoke multi-organ failure, including hemorrhage in the intestinal and respiratory systems and lymphoid necrosis. In this work, we analyzed the contribution of PB1-F2 in the pathology of chickens infected by a highly pathogenic AIV isolated in Niger in 2006 [34]. Unexpectedly, at low inoculum dose, the PB1-F2-expressing virus infection resulted in a reduced mortality in comparison to the ΔF2 virus. This feature contrasts with what is observed in mammals since PB1-F2 usually increases pathology in mice models [15], [17]–[19], [27], [34]. To characterize the functional causes of this observation, we explored the host responses of the infected chickens in two tissues: lung and blood. By comparing the genes differentially expressed in presence or absence of PB1-F2 during infection, we were able to identify genes signatures associated with this protein.

In lungs, PB1-F2 expression increases the transcription of genes involved in calcium signaling and alters the ER integrity to induce a ER-stress pathway. The ER stress response, also known as the unfolded protein response, is regulated by several enzymes including ERN1 (also known as IRE1). ERN1 activity has been shown to be important during the viral cycle of influenza virus [46]. The up-regulation of ERN1 in presence of PB1-F2 is related to the ability of PB1-F2 to misfold and aggregate in membrane environments [31]. The cellular Ca^2+^ concentration dynamics plays a critical role in epithelium homeostasis, and prolonged decrease of Ca^2+^ concentration within the ER triggers multiple cellular cascades that can ultimately lead to cell death [47]. A multitude of factors regulate the Ca^2+^ fluxes, including cytokines and reactive oxygen metabolites, but the strong membrane affinity of PB1-F2 and its capacity to alter membrane integrity [31], [48] supports the hypothesis that PB1-F2 itself could modify Ca^2+^ fluxes and then generate a gene signature in relation with “calcium signaling”. Importantly, Ueda and colleagues previously characterized the apoptosis induction of duck epithelial cells infected by a highly pathogenic H5N1 AIV through an extracellular Ca^2+^ influx mechanism [49]. This Ca^2+^ imbalance results in an excess of Ca^2+^ transport into the mitochondria, inducing a loss of mitochondrial membrane potential and apoptosis. Alternatively, the mitochondrial Ca^2+^ overload can occur through ER-mitochondria direct transfer, a mechanism facilitated by ER sensitization [50].

Interestingly, we identified a mitochondrial dysfunction gene signature associated to PB1-F2 expression in blood of infected-chickens. Mitochondrial dysfunction occurs when the ROS-mediated oxidative stress overpowers the antioxidant defense system. In parallel, inhibition of pathways relative to leukocyte activation was also evidenced in the presence of PB1-F2 (Fig. 8C). These two concomitant events are reminiscent of the fact that PB1-F2 was shown to inhibits immune response by decreasing the mitochondrial membrane potential [36]. Hence, PB1-F2 could trigger mitochondrial dysfunction in immune cells and may consequently deplete leukocytes through apoptosis induction in the infected host. This mechanism can be amplified through cytokine secretion such as CTLA-4 (Fig 4 and Fig 7). CTLA-4 is expressed by T-cells and has been described to play a crucial role in the immunomodulatory properties of these cells [51].

It is worth noting that the helicase RIG-I is absent in the chicken genome [52]. This is of first importance since PB1-F2 has been described to inhibit the type I interferon induction in mammals by binding to MAVS, the RIG-I adaptor protein allowing signal transduction [35], [36]. Thus, it seems unlikely that PB1-F2 acts in this way in the chicken host. In contrast to chickens, ducks express a functional form of RIG-I [52]. This differential expression of RIG-I between chickens and ducks is probably a key component that could explain the opposite PB1-F2 phenotype observed in these two species. Indeed, Schmolke and collaborators demonstrated that the deletion of PB1-F2 caused a delayed onset of pathologic signs and systemic spreading of virus [20]. These data in conjunction with previous findings in other studies support the hypothesis that the detrimental effect of PB1-F2 on the host could be mediated by the activated form of MAVS (*i.e.* filamentous form, [53]). The absence of RIG-I in the chicken genome imply that MAVS is probably less activated during infection and consequently reduce the negative effects of PB1-F2.

Essentially, no difference was observed in the outcome of the infection between the wt and the ΔF2 H5N1 virus using the dose of 1000 PFU. This would be consistent with the highly pathogenic genotype and phenotype of the virus due to multibasic cleavage site of the hemagglutinin. Interestingly, notable difference in the outcome of the infection was observed at an inoculation dose of 100 PFU, with the wt inoculated chickens being the survivors. Despite the molecular signatures associated with PB1-F2, it is still difficult to explain why the wt Nig06 is less virulent than the ΔF2 virus at a low dose. Analysis of gene regulation indicated that numbers of genes are strongly down regulated in the wt inoculated birds. While some of the genes are linked to pathogenesis, the overall combined effect of this lack of upregulation may have led to reduced mortality in this inoculation group of birds. It cannot be excluded that the role of PB1-F2 in birds is to attenuate the influenza virus to allow survival of the reservoir host, since almost all known avian isolates express this protein [22], [23], which appears to be excluded upon adaptation of the virus to a mammalian host, where gene upregulation caused by PB1-F2 contributes to pathogenesis [17], [19], [27], [28]. The down regulation of immune and inflammatory pathways in presence of PB1-F2 is associated to a better survival rate of infected chickens; this suggests that the host response could be implicated in the pathogenesis as previously described in mice models [54]. However, the molecular profiles of ΔF2-infected chickens and the cytokine analysis did not show aberrant cytokines production (data not shown) as described in the “cytokine storm” observed in human acute respiratory distress syndrome [55]. Nevertheless, a recent work by Kuchipudi and collaborators studied the kinetic of cell death induction in avian species infected with AIV and provided a link between the rapid induction of apoptosis and resistance to virus [56]. As a consequence, AIV could have evolved a mechanism implying PB1-F2 to delay the death of the host in order to have time to efficiently spread.

By comparing the host responses regulated by PB1-F2 in chicken and mouse using the same H5N1 wt- and mutant viruses, we were able to identify common and differentially regulated pathways in both hosts. The identified functions suggest that PB1-F2 expression provokes damages to the infected lungs in both species. PB1-F2 has affinity for hydrophobic environments and is able to disturb membrane integrity [31], [48], [57], this property is likely to induce damages to the epithelium structure and to alter ion channels control of fluid and electrolyte transport across epithelium. Consequently, the pathway “Aldosterone Signaling in Epithelial Cells” is regulated by PB1-F2 since it integrates genes implicated in ions flux and water retention in epithelia. Such genes like ASIC1, SCNN1A and SCNN1B (ENaCα and β) encode ion channels; their expressions are upregulated by PB1-F2 in chicken lungs (Fig. 9C) and suggest a perturbation of the electrodiffusion of the apical membrane of epithelial cells.

As a consequence of epithelial tissue integrity alteration, the wt Nig06 is able to spread more efficiently in infected embryos and also in intestine of infected chickens even if the biological relevance of the intestine viral replication data are questionable. Given that fecal-to-oral transmission is the most common mode of spread between birds, the aptitudes attributed to PB1-F2 to increase the biodispersion of the virus through the host organs could provide an advantage to avian viruses that express PB1-F2. This can explain why 96% of the avian virus genomes encode a functional PB1-F2. On the contrary, as PB1-F2 promotes inflammation in mammals [19], [27], [34] and facilitates secondary bacterial pneumonia [17], [21], the loss of PB1-F2 functions could be beneficial for an adaptation of the virus to the mammal hosts. This loss of function is particularly visible in the H1N1 influenza viruses isolated in humans: only 7% of the recently isolated viruses express a full length PB1-F2 [23].

In summary, the present study shed in light the complexity of PB1-F2 functions. PB1-F2 has strain specificity, cell type specificity and host specificity. Further research to identify relationship between specific PB1-F2 structural motif and pathogenesis is needed, a better understanding of the chicken antiviral responses is also necessary.

## Materials and Methods

### Ethics statement

All animal work was carried out in compliance with Canadian Council on Animal Care guidelines and was approved by the Animal Care Committee at the Canadian Science Centre for Animal and Human Health.

### Viruses

Influenza A/duck/Niger/2090/2006 (H5N1) was used in this study. Wild type (wt) and PB1-F2 knockout (ΔF2) viruses were produced by reverse genetics system using a bidirectional transcription plasmid derived from pHW2000 [58]. The viruses were prepared as previously described [34] and titrated on Madin Darby canine kidney (MDCK) cells.

### Embryos histological analysis

Ten days old embryos were fixed in 10% neutral phosphate buffered formalin, routinely processed and sectioned at the level of the brain, thorax, abdomen and legs as well as the chorioallantoic membrane. For immunohistochemistry, paraffin tissue sections were quenched for 10 minutes in aqueous 3% H_2_O_2_ then pretreated with proteinase K for 15 minutes. The primary antibody was a mouse monoclonal antibody specific for influenza A nucleoprotein (NP) (F26NP9, produced in-house) and was used at a 1∶10,000 dilution for one hour. They were then visualized using a horseradish peroxidase labelled polymer, Envision + system (anti-mouse) (Dako, USA), reacted with the chromogen diaminobenzidine (DAB). The sections were then counter stained with Gill's hematoxylin. Slides were examined and the extent of immunostaining was scored as weak (less than 20 cells staining), mild 1+ (< 25% of the section staining), moderate 2+ (25–50% of the section staining), extensive 3+ (51 to 75% of the section staining) or widespread 4+ (>75% of the section staining).

### Infection of chickens with wt or ΔF2 Nig06 and sample collection

Specific pathogen free (SPF) white Leghorn chickens at 40 days old were obtained from Fallowfield, Ottawa CFIA laboratory. They were floor housed in heated BSL3 animal cubicles and allowed 1 week of acclimatization before the start of experiments. Ten to 20 chickens per group were inoculated intranasally with 10^2^ or 10^3^ PFU per chicken of either wt or ΔF2 Nig06 H5N1 in 0.5 ml sterile PBS distributed into both nares for the survival experiments. Cloacal and oropharyngeal swabs were collected from each chicken on day 0 (prior to challenge) and at predetermined time points post challenge. Chickens were monitored daily for clinical signs including: depression, apathy, ruffled feathers, respiratory distress, hemorrhages and necrosis on the face, eyes, nares, combs, wattles, feet and legs. Clinical signs were scored as normal, mildly depressed, depressed and severely depressed. For animal welfare reasons, severely depressed chickens were humanely euthanized with intravenous injection of sodium pentobarbital (240 mg/ml). In second set of experiments targeting the host response, five chickens per group were inoculated intranasally with 10^3^ PFU per chicken of either wt or ΔF2 Nig06 H5N1 in 0.5 ml sterile PBS distributed into both nares. Blood was collected from the wing vein of anaesthetized chickens on days 0 and 2 post infection (pi). At 2 days pi, chickens were euthanized with intravenous injection of sodium pentobarbital (240 mg/ml), and tissue samples were collected. Swabs, trachea, lung, blood, heart, liver, kidney, spleen, brain, pancreas, intestine and thymus were stored at −70 °C.

### RNA extraction and qRT-PCR

RNA was isolated from swabs, bloods and lungs homogenates using the TRIzol-chloroform method according to the manufacturer's instructions (Invitrogen). RNA quality was checked on a Bioanalyzer 2100 (Agilent Technologies) and samples with a RNA Integrity Number (RIN) score between 7 and 9 were used in microarray or qRT-PCR experiments. Viral loads in swabs and tissues homogenates were determined by qRT-PCR assay specific for the influenza A M1 gene [59] with primers and probes that were modified to detect the Nig06 IAV: forward primer, 5′- CTT CTA ACC GAG GTC GAA ACG TA -3′; reverse primer, 5′-GGT GAC AGG ATC GGT CTT GTC TTT-3′; probe, 5′-TET-TCA GGC CCC CTC AAA GCC GAG-BHQ-3′ as previously described [34]. The mRNA levels of host genes were assayed using the Mastercycler realplex sequence detector (Eppendorf) and the double strand specific dye SYBR Green system (Applied Biosystems). Details of the primers are provided in Table 1. The PCR conditions and cycles were as follows: initial DNA denaturation 10 min at 95°C, followed by 40 cycles at 95°C for 15 sec, followed by an annealing step at 60°C for 15 sec, and then extension at 72°C during 30 sec. Each point was performed in triplicate. To ensure that the primers produced a single and specific PCR amplification product, a dissociation curve was performed at the end of the PCR cycle. Relative quantitative evaluation was performed by the comparative ΔΔCt method. The mean ΔCt obtained in mock-infected chickens for each gene was used as calibrator, after normalization to endogenous control β-actin. The results are presented as an n-fold difference relative to calibrator (RQ = 2^−ΔΔCt^).

**Table 1:** Primers used in real time PCR assays.

Gene name	Gene ID	Primer sequences		Tm	Product length
**STAT1**	424044	sense	GCAGGGAACAGAACAAATGAGG	62.23	129
		antisense	TGACAATGATGGGAAGTGATGTGG	63.47	
**β2M**	414830	sense	GAAGGCAGTTGCGCTGGTGG	64.56	158
		antisense	TGGTGATCCTGGGTGGGTGG	63.71	
**TLR4**	417241	sense	AATCCCAAACACCACCCTGG	60.18	198
		antisense	AGGTGCTGGAGTGAATTGGC	60.61	
**IFNAR1**	395665	sense	AGCAGAGAGGAATGCATCGG	59.89	91
		antisense	TCAAGACAACAGTCAGCGCA	60.18	
**CTLA-4**	424106	sense	TGCCGAAGTAATGGAAGTGACTC	63.01	149
		antisense	ATTTCAGTGAACTTGTCGCCTGTC	64.12	
**CCL5**	417465	sense	TCTCCATCCTCCTGGTTGCC	61.27	73
		antisense	CACACGGTTGTATCAGCCCC	61.02	
**TLR7**	418638	sense	ATCTTTCAGAGGTGGCTGCAC	60.61	120
		antisense	TTTTGGGAAACCAACGTCCT	57.85	
**HSPA2**	423504	sense	ATCATGTCTGGCAAAGGGCCG	63.34	172
		antisense	TGGCAGCATCCCCGATGAGG	64.50	
**IL2RG**	395199	sense	TCGTCAAGTACAAGAGCAACAAGG	63.58	179
		antisense	CCCAGAAGACAGGCACACTC	62.95	
**TLR6**	771173	sense	TCTGACGACATTTATCAAGGCA	57.80	107
		antisense	ATGGTCATCATCTCCCCAGA	57.50	
**BSL2L1**	373954	sense	GTTCGAGCATCCGACGTGAG	63.67	143
		antisense	ATTCACTACCTGCTCAAAGCTCTG	63.20	
**β-actin**	396526	sense	TTGCTGACAGGATGCAGAAG	60.14	141
		antisense	ACATCTGCTGGAAGGTGGAC	60.12	

### Microarray experiments

Transcriptional profiling was performed using the Agilent-026441 Gallus gallus Oligo Microarray (v2), 4×44Kslides (GEO accession: GPL15357). A dual color design was used to provide direct comparisons between infected lungs [infected-by-wt-virus/infected-by-ΔF2-virus], and between infected bloods and mock-infected bloods [infected-by-wt-virus/mock-infected] and [infected-by-ΔF2-virus/mock-infected]. To reduce potential experimental biases, RNA samples were collected from 5 different chickens for each experimental condition (Mock, wt-infected, ΔF2-infected). A total of 24 samples were analyzed, corresponding to 6 microarray slides. Fluorescently labeled cRNAs were obtained using the two colors Low Input Quick Amp Labeling Kit (Agilent Technologies) and starting from 100 ng of total RNA for each sample. supplemented with known synthetic RNA (two-color RNA spike-in kit, Agilent Technologies). cRNAs were subsequently purified using RNeasy Mini Spin columns (Qiagen) and the purified cRNAs were then run onto a Bioanalyzer 2100 using RNA 6000 Nano Chip (Agilent technologies). cRNA yields and specific activities were measured using a NanoDrop 2000 (Thermo Scientific). An equal amount of 800 ng of cyanine 3 and cyanine 5 labeled cRNA were hybridized for each sample. The hybridization and the washing steps were performed following the manufacturer's recommendations. The arrays were scanned using an Agilent G2505C scanner, and the scan protocol used a resolution of 5 µm and a 20 bit dynamic range. The resulting .tiff images were analyzed using the Feature Extraction software version 10.7.3.1 (Agilent) using the GE2_107_Sep09 protocol. For each channel, the median of the signal intensity was used without background subtraction. Data were analyzed to define genes that are differentially expressed between wt-infected and ΔF2-infected samples. Differentially expressed genes were identified with a False Discovery Rate (FDR) equal to 5%. Microarray data are available in the Gene Expression Omnibus (GEO) database (http://www.ncbi.nlm.nih.gov/geo/) under GEO accession number GSE56506.

### Transcriptomic analysis

For functional analysis the data files resulting from differential analysis were imported into GeneSpring GX 12.1 software (Agilent Technologies). Hierarchical clustering analysis was performed to analyze cellular genes that were differentially expressed during infection (Euclidian distance, average linkage). For further analysis, data files were uploaded into the Ingenuity Pathways Analysis (IPA) software (Ingenuity Systems).

### Statistical analysis

Survival of chickens was compared using Kaplan-Meier analysis and log-rank test. qRT-PCR quantification are expressed as the mean ± standard error of the mean (SEM) of at least four chickens, and statistical analyzes were performed using the paired Student T test. Correlation clustering and principal component analysis were performed on normalized data using the FactoMineR package. Ontological analysis made with IPA used the right-tailed Fisher's exact test to calculate a p-value determining the probability that each biological function and disease assigned to that data set is due to chance alone.

## Supporting Information

Figure S1 **Virus isolation by plaque assay on MDCK cells from oral swabs of chickens infected with 1000 PFU collected at 2 dpi (wt n = 15; ΔF2 n = 15) and at 4 dpi (wt n = 7; ΔF2 n = 5).** No live virus could be detected in swabs from 6 chickens in the wt-infected condition at day 2 pi, and from 9 chickens in the ΔF2-infected condition at day 2 pi.
(PDF)
