# Supplementary material for: PB1-F2 Attenuates Virulence of Highly Pathogenic Avian H5N1 Influenza Virus in Chickens
Source: PLoS One. 2014 Jun 24;9(6):e100679. doi: 10.1371/journal.pone.0100679 (PMC4069075; doi:10.1371/journal.pone.0100679)
Supplement: Figure S1 — Virus isolation by plaque assay on MDCK cells from oral swabs of chickens infected with 1000 PFU collected at 2 dpi (wt n = 15; ΔF2 n = 15) and at 4 dpi (wt n = 7; ΔF2 n = 5). No live virus could be detected in swabs from 6 chickens in the wt-infected condition at day 2 pi, and from 9 chickens in the ΔF2-infected condition at day 2 pi. (PDF) [file pone.0100679.s001.pdf]

Figure S1. Leymarie *et al.*

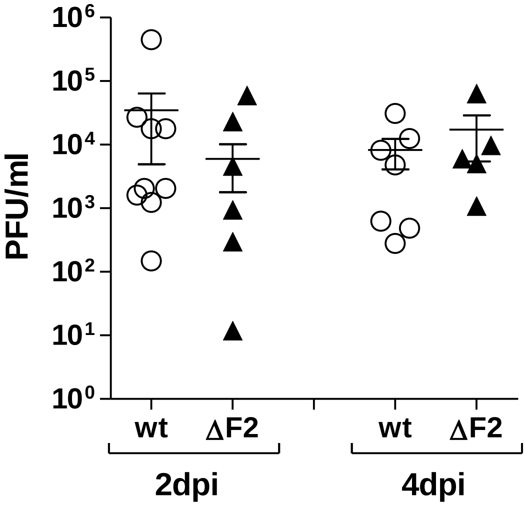

Supplemental Figure S1:

Virus isolation by plaque assay on MDCK cells from oral swabs of chickens infected with 1000 PFU collected at 2 dpi (wt n=15; ΔF2 n=15) and at 4 dpi (wt n=7; ΔF2 n=5). No live virus could be detected in swabs from 6 chickens in the wt-infected condition at day 2 pi, and from 9 chickens in the ΔF2-infected condition at day 2 pi.
